# Usability and Behavioral Intention in Quasimandatory National Digital Health Systems: The Mediating Role of E‐Satisfaction

**DOI:** 10.1155/jonm/7864014

**Published:** 2026-05-16

**Authors:** Burhan Kılıç, Yakup Durmaz

**Affiliations:** ^1^ Independent Researcher, Gaziantep, Turkey; ^2^ Department of Marketing, Kilis 7 Aralık University, Faculty of Economics and Administrative Sciences-Kilis, Kilis, Turkey, kilis.edu.tr

**Keywords:** behavioral intention, e-Nabız, mobile health, satisfaction, usability

## Abstract

The rapid expansion of national digital health infrastructures has transformed the way citizens interact with healthcare systems. However, the long‐term success of these platforms depends not only on technological implementation but also on users’ perceptions and continued engagement. This study investigates how the usability of a national mobile health application influences users’ behavioral intention through the mediating role of e‐satisfaction. Drawing on usability and technology acceptance literature, the study proposes a research model in which the perceived usability of a mobile health application positively affects users’ behavioral intention both directly and indirectly via e‐satisfaction. Empirical data were collected from users of the Turkish national digital health platform e‐Nabız through a structured survey. The proposed research model was tested using structural equation modeling techniques. The results indicate that mobile health application usability significantly enhances users’ e‐satisfaction, which in turn positively influences behavioral intention, including recommendation intention and continued preference intention. Interestingly, the analysis revealed that even negative usability perceptions were associated with increased e‐satisfaction, likely reflecting the quasimandatory nature of the platform, where functional necessity and perceived value can outweigh usability difficulties. The findings also demonstrate the mediating role of e‐satisfaction in the relationship between usability and behavioral intention. By focusing on a large‐scale national digital health platform, this study contributes to the growing literature on digital health adoption by highlighting the importance of usability‐driven user experience in quasimandatory digital health systems. The results provide practical insights for policymakers and digital health system designers seeking to improve citizen engagement and long‐term utilization of national digital health infrastructures.

## 1. Introduction

Digital transformation has become a structural imperative for contemporary health systems. Global organizations such as the World Health Organization (WHO) and the International Telecommunication Union (ITU) emphasize that interoperable national digital health infrastructures are essential for achieving universal health coverage and sustainable health system development [1, 2]. The WHO–ITU Digital Health Platform Handbook conceptualizes national digital health platforms as scalable, reusable, and interoperable infrastructures that enable integrated service delivery across healthcare actors [2]. In alignment with these global strategies, many countries have launched nationwide digital health missions aimed at standardizing health records, strengthening interoperability, and building integrated digital public ecosystems [3]. These initiatives reflect a shift from fragmented eHealth applications toward centralized, policy‐driven digital health infrastructures embedded within national governance frameworks.

Although prior research has extensively examined the architectural, interoperability, and governance dimensions of national digital health systems [1, 3], significantly less attention has been devoted to citizen‐level behavioral dynamics following system deployment. Technical implementation alone does not guarantee long‐term public value creation. The sustained effectiveness of national digital health infrastructures depends on users’ ongoing engagement, perceived usefulness, and satisfaction beyond initial adoption.

Postadoption research in information systems has largely relied on expectation–confirmation theory (ECT) and technology continuance models to explain sustained system use [4, 5]. Empirical applications predominantly examine voluntary digital services such as mobile banking, e‐learning, social media, and mobile commerce platforms [6–8]. Within these voluntary contexts, users retain autonomy to discontinue usage, and satisfaction consistently functions as a central mediator between perceived system characteristics and continuance intention [9]. Hedonic motivation, habit, and performance expectancy frequently emerge as significant predictors of sustained engagement [10].

However, national digital health infrastructures frequently operate within structurally different environments. Unlike commercial mobile applications, public‐sector digital systems may be institutionally embedded within regulatory and administrative frameworks that constrain user discretion. Research on mandatory information systems suggests that established success mechanisms may function differently when exit options are limited [11]. Studies extending the DeLone and McLean information systems success model to mandatory government contexts indicate that perceived usefulness often outweighs technical system quality in explaining user satisfaction [11, 12]. These findings imply that utilitarian value becomes more salient than experiential attributes when system usage is structurally embedded.

Despite these insights, research remains fragmented at the intersection of (1) national digital health infrastructures, (2) postadoption continuance mechanisms, and (3) quasimandatory public digital ecosystems. Mandatory system studies typically focus on internal government information systems used by public employees [11], whereas citizen‐facing digital health platforms represent a distinct hybrid context combining public governance structures with individual‐level behavioral processes. Conversely, continuance intention research has primarily examined voluntary consumer technologies [4, 5], leaving open questions regarding whether satisfaction‐mediated mechanisms retain explanatory power in structurally embedded public health infrastructures.

Against this background, the present study investigates the postadoption engagement mechanisms of Turkey’s national digital health platform, e‐Nabız, operating within a quasimandatory public health infrastructure. Specifically, the study examines how positive and negative usability perceptions influence behavioral intentions—conceptualized as recommendation intention and continued preference intention—through the mediating role of e‐satisfaction. By empirically testing this mechanism within a national digital health ecosystem, the study evaluates whether satisfaction‐based postadoption models remain robust in structurally embedded public digital environments. In doing so, it contributes to the literature by extending continuance research beyond voluntary consumer applications and by contextualizing usability‐driven satisfaction dynamics within a quasimandatory digital infrastructure.

## 2. Theoretical Background and Research Hypotheses

### 2.1. The Case of e‐Nabız: A Quasimandatory National Digital Health Platform

e‐Nabız is Turkey’s national personal health record (PHR) system that enables citizens to access medical examinations, diagnoses, prescriptions, laboratory results, and treatment records generated within healthcare institutions nationwide since its nationwide implementation in 2015 [13]. The platform operates via web‐based and mobile interfaces, allowing individuals to view and manage their consolidated health data and to share selected information with healthcare professionals [14].

Beyond passive record viewing, the system allows users to manually enter personal health parameters such as blood pressure, glucose levels, weight, and pulse, facilitating longitudinal self‐monitoring. From a health management perspective, the platform aims to enhance patient engagement, promote informed participation in treatment decisions, and reduce redundant medical procedures by enabling access to retrospective and real‐time health information [15].

Importantly, e‐Nabız operates within a centralized national health data ecosystem in which healthcare providers are required to transfer patient data to the Ministry of Health systems. As a result, citizens’ health records are generated automatically, regardless of whether they are active on the platform. While individuals are not legally obligated to use the interface, comprehensive access to consolidated national health records is structurally embedded within the system. This configuration situates e‐Nabız within a quasimandatory digital health environment, making it theoretically distinct from purely voluntary mobile health (mHealth) applications.

Within this context, usability perceptions may play a critical role in shaping satisfaction and subsequent behavioral intention, even when system participation is institutionally embedded rather than purely discretionary.

### 2.2. Usability in Quasimandatory Digital Health Systems

Usability is defined by ISO 9241‐11 as “the extent to which a product can be used by specified users to achieve specified goals with effectiveness, efficiency, and satisfaction in a specified context of use.” A systematic review indicates that approximately 88% of mobile application usability studies adopt this definition, reflecting its conceptual dominance in digital health research [16]. Similarly, usability has been described as the overall quality of a user’s interaction experience, encompassing effectiveness, efficiency, and satisfaction [17].

The rapid expansion of mHealth technologies has intensified the importance of usability as a system quality attribute. Earlier evidence reported that more than 165,000 mHealth applications were available in digital marketplaces, with widespread adoption among adult populations. The growth of mHealth has been driven by the proliferation of smartphones, increased digital literacy, and the portability of mobile technologies, enabling individuals to access health information anytime and anywhere [18].

Beyond adoption statistics, empirical research highlights the functional and psychological consequences of usability in health contexts. Studies indicate that users frequently experience difficulties related to interface complexity, inconsistent system design, foreign language barriers, and limited health literacy [19]. For example, Goswami et al. [19], using the MAUQ instrument to evaluate a mHealth application for Type 2 diabetes management, found that usability perceptions were not significantly differentiated by demographic variables, suggesting that interaction experience itself, rather than user characteristics, plays a central evaluative role. Moreover, digital health applications that provide accessible and comprehensible information have been associated with improved psychological outcomes, such as reduced anxiety and depression scores among breast cancer patients [20]. These findings suggest that usability in health systems influences not only operational efficiency but also users’ broader evaluative and emotional responses.

Within information systems research, usability has traditionally been linked to technology acceptance frameworks. The technology acceptance model [21] conceptualizes perceived ease of use as a determinant of attitudes and behavioral intention, primarily in voluntary adoption settings. However, in quasimandatory digital infrastructures—such as centralized national PHR systems—usability may function differently. In these systems, health data are automatically generated and stored regardless of active interface engagement. Although individuals are not legally compelled to use the interface, structural embedding reduces complete exit options. Consequently, usability perceptions are expected to shape evaluative judgments about the system rather than determine initial adoption decisions.

User experience literature further suggests that usability perceptions are not unidimensional. During system interaction, users simultaneously form positive and negative evaluations. Positive use reflects clarity of system functions, ease of learning, confidence during interaction, and effective integration of features. Negative use captures perceptions of complexity, inconsistency, confusion, or technical difficulty. Distinguishing between these dual dimensions allows for a more nuanced understanding of interaction quality, particularly in environments where engagement intensity and voluntary interface use vary despite institutional embedding.

From a postadoption perspective, satisfaction operates as the central evaluative mechanism translating system‐related perceptions into behavioral responses [4, 5]. Accordingly, within quasimandatory national digital health systems, usability is expected to influence users’ e‐satisfaction through both its positive and negative dimensions. Therefore, the following hypotheses are proposed:

 H1a: Positive use positively influences e‐satisfaction.
 H1b: Negative use negatively influences e‐satisfaction.

### 2.3. e‐Satisfaction as a Postadoption Evaluation Mechanism

User satisfaction is traditionally defined as the evaluative judgment that results from comparing perceived performance with prior expectations [22]. In marketing literature, satisfaction reflects customers’ overall assessment of whether a product or service meets their needs and expectations [23, 24]. Over time, satisfaction has been recognized as a central determinant of customer retention and loyalty, shaping long‐term relational outcomes [25].

With the expansion of digital environments, the concept evolved into e‐satisfaction, referring to users’ affective and cognitive evaluation of their online experience. Early academic investigations conceptualized e‐satisfaction as users’ contentment with online interactions, particularly in e‐commerce platforms ([26], as cited in [27]). Subsequent studies defined e‐satisfaction as consumers’ evaluation of their browsing or purchasing experiences in digital environments [28–31]. It has also been described as the accumulated satisfaction derived from repeated online interactions over time [32, 33].

While these definitions primarily originate from e‐commerce contexts, digital health infrastructures differ in several important respects. Unlike commercial platforms, national digital health systems involve sensitive medical data, institutional governance, and public service logic. Users do not merely evaluate transactional efficiency; they assess information clarity, system reliability, privacy assurance, and perceived health‐related value. Therefore, e‐satisfaction in digital health environments reflects a broader evaluative mechanism encompassing trust, informational adequacy, and experiential quality.

ECT provides a theoretical foundation for understanding satisfaction in postadoption contexts [22]. According to ECT, users form expectations before system use and subsequently evaluate actual performance. When perceived performance confirms or exceeds expectations, satisfaction emerges; when it falls short, dissatisfaction occurs. In information systems research, continuance models adapted from ECT identify satisfaction as the primary determinant of postadoption intention [4, 5]. Thus, satisfaction functions as the psychological mechanism translating system‐related perceptions into behavioral responses.

In quasimandatory digital health infrastructures, satisfaction may play a distinctive yet critical role. Although complete system abandonment may not be feasible due to structural embedding, variations in engagement intensity, voluntary interface usage, and willingness to recommend the platform remain behaviorally meaningful. In such contexts, e‐satisfaction does not merely determine whether users adopt the system; rather, it influences how actively and positively they engage with it.

Accordingly, within national digital health platforms, users who evaluate their digital interaction experience favorably are expected to demonstrate stronger behavioral intentions, both in terms of recommending the system and expressing continued preference for its use. Therefore, the following hypotheses are proposed:

 H2a: E‐satisfaction positively influences recommendation intention.
 H2b: E‐satisfaction positively influences continued preference intention.

### 2.4. Behavioral Intention in Structurally Embedded Digital Health Platforms

Behavioral intention refers to an individual’s conscious plan or willingness to perform a particular behavior in the future [34]. Within marketing and information systems literature, behavioral intention has frequently been conceptualized as a predictor of actual behavior, including repurchase, continued usage, and recommendation [35, 36].

In traditional voluntary consumption contexts, behavioral intention often reflects repurchase intention, revisit intention, or willingness to recommend a product or service [37–40]. Recommendation intention, in particular, has become increasingly significant in digital environments where social communication channels amplify user opinions [41, 42]. Positive word‐of‐mouth in digital platforms contributes not only to system legitimacy but also to network‐based value creation.

However, behavioral intention in national digital health infrastructures differs from purely commercial contexts. In voluntary digital services, discontinuance remains a realistic option when dissatisfaction arises. In contrast, structurally embedded public systems—such as centralized PHR platforms—limit complete exit because health data generation occurs independently of active interface usage. Consequently, behavioral intention in quasimandatory systems does not necessarily capture adoption decisions but rather reflects the intensity and quality of engagement.

Within this framework, behavioral intention is conceptualized through two complementary dimensions. First, recommendation intention represents users’ willingness to advocate for the system to others, signaling trust and perceived value. Second, continued preference intention reflects the tendency to prioritize and voluntarily use the platform when interacting with health information in the future. While users may not be able to abandon the broader ecosystem, they retain discretion over interface interaction, feature utilization, and engagement depth.

Thus, in quasimandatory digital health environments, behavioral intention captures meaningful variations in user engagement rather than mere system presence. This reconceptualization aligns behavioral intention with postadoption theory, where continued usage and advocacy serve as indicators of system success.

### 2.5. The Mediating Role of e‐Satisfaction

Postadoption research in information systems consistently suggests that system‐related perceptions influence behavioral outcomes primarily through evaluative judgments rather than direct causal paths. Within ECT [22], satisfaction emerges as the central mechanism through which perceived performance translates into continuance intention. Similarly, continuance models adapted to digital contexts emphasize that system characteristics shape behavioral intention indirectly via satisfaction [4, 5].

Building on general usability research insights, the present study adopts a postadoption perspective and emphasizes e‐satisfaction as the central mechanism linking usability perceptions to behavioral intention in quasimandatory digital health systems, without formally integrating TAM or ECM models.

Within quasimandatory national digital health systems, users cannot fully avoid data integration within the broader ecosystem; however, they retain discretion over interface engagement, feature utilization, and active participation. In this context, satisfaction functions as a psychological evaluation filter. Positive interaction experiences enhance users’ affective evaluation of the platform, which in turn strengthens their willingness to recommend and continue prioritizing its use. Conversely, negative interaction experiences may diminish evaluative judgments, thereby reducing engagement‐related intentions.

Testing the mediating role of e‐satisfaction is therefore theoretically meaningful. If usability influences behavioral intention primarily through satisfaction, this would indicate that traditional postadoption mechanisms remain operative even under structurally embedded conditions. Conversely, a weak or absent mediation effect would suggest that quasimandatory infrastructures alter established continuance dynamics.

Accordingly, this study proposes that the effects of positive and negative usability perceptions on behavioral intention operate indirectly through e‐satisfaction. Therefore, the following hypotheses are proposed:

 H3a: E‐satisfaction mediates the relationship between positive use and recommendation intention.
 H3b: E‐satisfaction mediates the relationship between positive use and continued preference intention.
 H3c: E‐satisfaction mediates the relationship between negative use and recommendation intention.
 H3d: E‐satisfaction mediates the relationship between negative use and continued preference intention.

**FIGURE 1:**
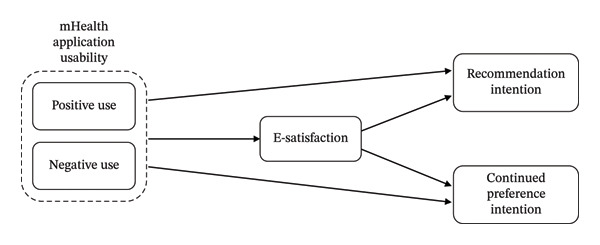
Research model.

Figure 1 presents the research model of the current study. Grounded in postadoption theory and situated within a quasimandatory national digital health context, the model examines how positive and negative usability perceptions shape e‐satisfaction, which in turn influences behavioral intention through recommendation and continued preference intentions.

## 3. Method

### 3.1. Participants and Procedures

This study adopted a quantitative, cross‐sectional survey design to examine the relationships between mHealth application usability, e‐satisfaction, and behavioral intention in the context of the e‐Nabız system in Turkey. As of 2026, e‐Nabız has reached approximately 78.5 million registered users, making it one of the largest nationally integrated digital health platforms worldwide [43]. Unlike voluntary mHealth applications, e‐Nabız operates as a centralized public digital health infrastructure embedded within Turkey’s healthcare delivery system. Given its large‐scale implementation and quasimandatory use for accessing health services, investigating user perceptions within this context provides important theoretical and policy‐relevant insights for national digital health governance and mandatory digital service environments.

The population of the study consisted of individuals aged 18 and over who actively use the e‐Nabız application in Turkey. Sample size adequacy was first determined using the population‐sample table developed by Yazıcıoğlu and Erdoğan [44], which indicates that a minimum sample of 384 respondents is sufficient to represent large populations (100,000–100 million) at a 95% confidence level. In addition, an a priori power analysis conducted using G ∗ Power *f* ^2^ = 0.05, *α* = 0.05, power = 0.90, five predictors [45] suggested a minimum required sample of 335 participants. The final sample of 889 respondents substantially exceeded both thresholds, ensuring robust statistical power. A total of 1000 e‐Nabız users were invited to participate in the study through an online survey administered by the author. Of these, 902 responses were received. After excluding 13 incomplete questionnaires, 889 valid responses were retained, yielding an effective response rate of 88.9%.

Convenience sampling was employed due to practical accessibility to e‐Nabız users across different regions of Turkey. Although this approach enabled access to a large and diverse user base, it may limit the generalizability of the findings. Given the voluntary nature of participation, self‐selection bias may have occurred, as individuals with stronger attitudes toward the application could have been more likely to respond. To assess potential nonresponse bias, early and late respondents were compared in terms of demographic characteristics, and no significant differences were found, suggesting minimal bias.

Data were collected between March 01, 2022, and June 30, 2022. Ethical approval was obtained from the Hasan Kalyoncu University Ethics Committee (18.02.2022; No.: 10246). Participation was voluntary and anonymous. Respondents were informed that there were no right or wrong answers and that data would be used solely for academic purposes, thereby reducing potential common method bias (CMB).

The measurement items were translated and back‐translated by two independent bilingual experts to ensure linguistic and conceptual equivalence. Cultural considerations related to healthcare service usage in Turkey were carefully addressed. A pilot study was conducted with 50 e‐Nabız users to assess clarity and comprehensibility. The pilot data were not included in the final analysis.

The demographic characteristics of the sample are presented in Table 1. Of the participants, 52.3% were female, and 47.7% were male. In terms of marital status, 33.6% were married, and 66.4% were single. Regarding age distribution, 48.8% were between 18 and 25 years, 33.3% between 26 and 35 years, 14.7% between 36 and 45 years, and 3.1% were 46 years or older. Educational attainment ranged from primary education (1.7%) to doctoral level (3.9%), with the majority holding associate (38.8%) or bachelor’s degrees (35.1%).

**TABLE 1:** Demographic information.

Variables	Groups	*N*	%
Gender	Female	465	52.3
	Male	424	47.7

Civil status	Married	299	33.6
	Single	590	66.4

Age	18–25	434	48.8
	26–35	296	33.3
	36–45	131	14.7
	46 and above	28	3.1

Education status	Primary‐middle school	15	1.7
	High school	124	13.9
	Associate degree	345	38.8
	Bachelor’s degree	312	35.1
	Master	58	6.5
	PhD	35	3.9

Concerning usage patterns, 50.5% of respondents reported using e‐Nabız for all available functions, 17.2% primarily for appointment scheduling, 19.8% to view test results, 12.0% to access historical medical records, and 0.4% for medication reminders. In terms of frequency, 26.9% reported using the application once a month, 21.9% several times a month, 21.6% several times a year, 8.7% once a year, 7.6% several times a week, 7.4% once a week, 3.4% once a day, and 2.5% several times a day (see Table 2).

**TABLE 2:** Other information about participants.

Variables	Groups	*N*	%
Reason of e‐Nabız use	To make an appointment	153	17.2
	To see the test results	176	19.8
	For medication reminders	4	0.4
	To access my historical data	107	12.0
	All of them	449	50.5

Frequency of e‐Nabız use	Once a day	30	3.4
	Several times a day	22	2.5
	Once a week	66	7.4
	Several times a week	68	7.6
	Once a month	239	26.9
	Several times a month	195	21.9
	Once a year	77	8.7
	Several times a year	192	21.6

### 3.2. Measures

All constructs were measured using previously validated scales adapted to the Turkish context. Unless otherwise stated, responses were recorded on a five‐point Likert scale ranging from 1 (*strongly disagree*) to 5 (*strongly agree*). All constructs were specified as reflective. To reduce the potential risk of CMB, several procedural remedies were applied during the survey design stage, such as varying the order of the questions and ensuring respondent anonymity [46, 47]. In addition, to verify the randomness of the collected data, a multivariate runs test was conducted, and the results are reported in the analysis section. The measurement instruments employed in the study are described below.

#### 3.2.1. mHealth Application Usability

mHealth application usability was assessed using the System Usability Scale (SUS) originally developed by Brooke [48] and later adapted into Turkish by Demirkol and Şeneler [49]. Consistent with the Turkish adaptation, the construct was modeled with two dimensions: positive use and negative use, each consisting of five items. The positive use dimension captures perceived ease of use, integration, and confidence in interacting with the application. A sample item is “I think this application is easy to use.” The negative use dimension reflects perceived complexity and usability difficulties, with items such as “I found this application very cumbersome to use.” Internal consistency values exceeded the recommended threshold of 0.70, indicating satisfactory reliability. Detailed reliability and validity statistics are reported in Appendix 1.

Although the original SUS was conceptualized as a unidimensional measure, subsequent research has demonstrated that the scale can exhibit a two‐factor structure, distinguishing between positive and negative usability perceptions. Consistent with the validated Turkish adaptation by Demirkol and Şeneler [49], this study retained the two‐dimensional structure to capture both facilitating and inhibiting aspects of usability. Preserving this distinction allows for a more nuanced examination of how favorable and unfavorable usability experiences may differentially influence e‐satisfaction and behavioral intention in a large‐scale public digital health context.

#### 3.2.2. e‐Satisfaction

E‐satisfaction was measured using the scale adapted and validated by Bayram and Şahbaz [50]. The scale captures users’ overall affective and cognitive evaluation of their service experience through the digital platform. The construct consists of four items reflecting satisfaction and decision affirmation. A representative item is “I feel good about my decision to use this application.” Reliability and validity indicators demonstrated strong internal consistency and convergent validity (see Appendix 1).

#### 3.2.3. Behavioral Intention

Behavioral intention was measured using the instrument adapted and validated in Turkish by Hoşgör [51]. The construct was operationalized with two subdimensions: recommendation intention and continued preference intention. Recommendation intention reflects users’ willingness to advocate the application to others (e.g., “If asked for advice, I would recommend this application”). Continued preference intention captures future usage commitment (e.g., “If I had to make the choice again, I would choose this application”). Both subdimensions demonstrated strong internal consistency and satisfactory construct validity (see Appendix 1).

## 4. Results

Before hypothesis testing, several preliminary analyses were conducted to ensure the adequacy and validity of the data. Given that all variables were collected using a self‐report survey at a single point in time, CMB was assessed using Harman’s single‐factor test [47]. The unrotated exploratory factor analysis revealed that the first factor accounted for 28.6% of the total variance, which is below the recommended threshold of 50%, indicating that CMB was not a serious concern. Sampling adequacy was evaluated using the Kaiser–Meyer–Olkin (KMO) measure and Bartlett’s test of sphericity. The KMO value was 0.921 (*p* < 0.01), indicating excellent sampling adequacy and confirming that the data were suitable for factor analysis [52].

The hypothesized relationships were tested using structural equation modeling (SEM) with maximum likelihood estimation in AMOS Version 24 [53]. SEM was selected due to its ability to simultaneously assess measurement validity and structural relationships among latent constructs. This technique assumes normally distributed data, the absence of significant outliers, and no missing values [54, 55]. All skewness and kurtosis values were within the acceptable range of −1.5–+1.5, supporting the assumption of normality [56].

A two‐step analytical procedure was followed in accordance with Anderson and Gerbing [57]. First, confirmatory factor analysis (CFA) was conducted to validate the measurement model. Second, the structural model was evaluated to test the hypothesized direct and indirect relationships. To assess the mediation effect, bootstrapping with 5000 resamples and a 95% confidence interval was performed. Bootstrapping enhances the robustness and stability of parameter estimates, particularly for indirect effects [58].

### 4.1. CFA

Following the recommendations of Hair et al. [58], we employed CFA because the measurement instruments were adapted from prior research. Multiple models were evaluated using commonly accepted fit indices (see Table 3), normed chi‐square (*χ* ^2^/df ≤ 3.0), goodness‐of‐fit index (GFI ≥ 0.90), comparative Fit Index (CFI ≥ 0.90), root mean square error of approximation (RMSEA ≤ 0.08), and root mean residual (RMR ≤ 0.08), with all factor loadings exceeding 0.40 [58]. After allowing covariances between selected error terms, the five‐factor model demonstrated an adequate fit to the data, with *χ* ^2^/df = 2.672 (531.728/199), CFI = 0.973, SRMR = 0.033, AGFI = 0.945, and RMSEA = 0.043, all meeting the recommended thresholds.

**TABLE 3:** Comparison of measurement models.

	*χ* ^2^	df	*χ* ^2^/df	CFI	SRMR	AGFI	RMSEA
1‐ Five‐factor model	531.728	199	2.627	0.973	0.033	0.945	0.043
1‐ Four‐factor model	729.680	202	3.612	0.951	0.040	0.913	0.054
1‐ Three‐factor model	833.159	204	4.084	0.942	0.042	0.897	0.059
2‐ Two‐factor model	870.950	205	4.249	0.938	0.048	0.893	0.060
3‐ One‐factor model	914.302	205	4.460	0.934	0.048	0.886	0.062

*Note:* 1 = positive use, negative use, e‐satisfaction, recommendation intention, continued preference intention. 2 = positive use + negative use, e‐satisfaction, recommendation intention, continued preference intention. 3 = positive use + negative use + e‐satisfaction andrecommendation intention, continued preference intention. 4 = positive use + negative use + e‐satisfaction andrecommendation intention + continued preference intention. 5 = positive use + negative use + e‐satisfaction + recommendation intention + continued preference intention.

### 4.2. Validity and Reliability

The reliability and validity of the measurement model were assessed using established criteria in the SEM literature. Internal consistency was examined through Cronbach’s alpha and composite reliability (CR). All Cronbach’s alpha values exceeded the recommended threshold of 0.70, and all CR values were above 0.60, indicating satisfactory reliability [59]. Convergent validity was evaluated using average variance extracted (AVE). All AVE values were greater than 0.50, meeting the recommended threshold and supporting convergent validity. Discriminant validity was assessed by comparing AVE and maximum shared variance (MSV). In all cases, MSV values were lower than the corresponding AVE values, indicating adequate discriminant validity [60–62]. Additionally, the heterotrait–monotrait (HTMT) ratio was examined, and all HTMT values were below the conservative threshold of 0.90, further confirming construct distinctiveness [63]. Overall, the findings demonstrate that the measurement model exhibits satisfactory reliability, convergent validity, and discriminant validity. Detailed statistics are presented in Appendix 1 Table A1 and 4.

**TABLE 4:** Heterotrait–monotrait (HTMT) ratio analysis.

Variables	1	2	3	4
1‐ Positive use				
2‐ Negative use	0.623			
3‐ E‐satisfaction	0.840	0.567		
4‐ Recommendation intention	0.728	0.504	0.849	
5‐ Continued preference intention	0.734	0.461	0.781	0.846

### 4.3. Descriptive and Correlation Analysis

Descriptive statistics and correlation coefficients for all study variables are presented in Table 5. The mean values ranged between 3.62 and 3.86, indicating generally positive evaluations of usability, satisfaction, and behavioral intentions among e‐Nabız users. Correlation analysis revealed significant and positive relationships among the main constructs. Positive use was positively associated with e‐satisfaction (*r* = 0.688, *p* < 0.01) and behavioral intention dimensions. Similarly, negative use showed significant associations with e‐satisfaction (*r* = 0.444, *p* < 0.01) and behavioral intention. E‐satisfaction was strongly correlated with recommendation intention (*r* = 0.763, *p* < 0.01) and continued preference intention (*r* = 0.688, *p* < 0.01). All correlations were below the threshold of 0.90, suggesting no multicollinearity concerns. Overall, the direction and significance of the correlations provide preliminary support for the hypothesized relationships, warranting further examination through structural model analysis.

**TABLE 5:** Relationships between variables.

Variables	Mean	SE	1	2	3	4	5
1‐ Positive use	3.624	0.696	1				
2‐ Negative use	3.719	0.692	0.462^∗∗^	1			
3‐ E‐Satisfaction	3.856	0.710	0.688^∗∗^	0.444^∗∗^	1		
4‐ Recommendation intention	3.803	0.722	0.603^∗∗^	0.398^∗∗^	0.763^∗∗^	1	
5‐ Continued Preference intention	3.810	0.711	0.601^∗∗^	0.363^∗∗^	0.688^∗∗^	0.755^∗∗^	1

^∗∗^ *p* < 0.01.

### 4.4. Hypotheses Testing

The hypothesized relationships were tested using SEM with maximum likelihood estimation in AMOS 24. The results of the structural model are presented in Table 6.

**TABLE 6:** Results of the structural model.

Hypotheses	*β*	CR	SE	*p*
Positive use⟶e‐satisfaction	0.867	18.728	0.046	0.001
Positive use⟶recommendation intention	−0.008	−0.114	0.070	0.838
Positive use⟶continued preference intention	0.280	3.553	0.079	0.038
e‐satisfaction⟶recommendation intention	0.860	11.704	0.073	0.001
e‐satisfaction⟶continued preference intention	0.560	7.261	0.077	0.001
Negative use⟶e‐satisfaction	0.807	9.487	0.085	0.001
Negative use⟶recommendation intention	0.065	1.328	0.049	0.307
Negative use⟶continued preference intention	0.036	0.635	0.057	0.729

As shown in Table 6, positive use had a significant and positive effect on e‐satisfaction (*β* = 0.867, *p* < 0.001). Therefore, H1a was supported. However, contrary to expectations, negative use had a significant positive effect on e‐satisfaction (*β* = 0.807, *p* < 0.001), whereas the hypothesis predicted a negative relationship. Thus, H1b was not supported. Furthermore, e‐satisfaction had a significant positive effect on recommendation intention (*β* = 0.860, *p* < 0.001) and continued preference intention (*β* = 0.560, *p* < 0.001). These findings support H2a and H2b, indicating that higher levels of e‐satisfaction lead to stronger behavioral intentions toward the application.

To test the mediating role of e‐satisfaction, a bootstrapping procedure with 5000 resamples and a 95% confidence interval was conducted. The mediation results are reported in Table 7.

**TABLE 7:** Results for mediation.

Hypotheses	*β*	CR	SE	*p*	Bootstraps 95%	
					LLCI	ULCI
*E-satisfaction mediates the relationship between positive use and recommendation intention.*						
Direct path	−0.008	−0.114	0.070	0.838	−0.214	0.118
Indirect path	0.736	—	0.094	0.003	0.618	0.943

*E-satisfaction mediates the relationship between positive use and continued preference intention.*						
Direct path	0.280	3.553	0.079	0.038	0.076	0.492
Indirect path	0.473	—	0.116	0.011	0.285	0.667

*E-satisfaction mediates the relationship between negative use and recommendation intention.*						
Direct path	0.065	1.328	0.049	0.307	−0.027	0.117
Indirect path	0.488	—	0.045	0.002	0.429	0.590

*E-satisfaction mediates the relationship between negative use and continued preference intention.*						
Direct path	0.036	0.635	0.057	0.729	−0.062	0.101
Indirect path	0.454	—	0.047	0.002	0.395	0.557

First, the mediating role of e‐satisfaction in the relationship between positive use and recommendation intention was examined. The direct path was insignificant (*β* = −0.008, *p* = 0.838), whereas the indirect path was significant (*β* = 0.736, *p* = 0.003) without a zero value between the lower (i.e., 0.618) and upper limits (i.e., 0.943). Therefore, H3a was supported. Second, the mediating role of e‐satisfaction between positive use and continued preference intention was tested. Both the direct path (*β* = 0.280, *p* = 0.038) and the indirect path (*β* = 0.473, *p* = 0.011) were significant without a zero value between the lower (i.e., 0.285) and upper limits (i.e., 0.667). Thus, H3b was supported. Third, the mediating role of e‐satisfaction between negative use and recommendation intention was examined. While the direct path was insignificant (*β* = 0.065, *p* = 0.307), the indirect path was significant (*β* = 0.488, *p* = 0.002) without a zero value between the lower (i.e., 0.429) and upper limits (i.e., 0.590). Accordingly, H3c was supported. Finally, the mediating role of e‐satisfaction between negative use and continued preference intention was analyzed. The direct path was insignificant (*β* = 0.036, *p* = 0.729), whereas the indirect path was significant (*β* = 0.454, *p* = 0.002) without a zero value between the lower (i.e., 0.395) and upper limits (i.e., 0.557). Therefore, H3d was supported.

Overall, the results indicate that e‐satisfaction plays a significant mediating role in the relationship between mHealth application usability and behavioral intention.

## 5. Discussion and Implications for Theory

This study contributes to the growing body of research on digital health technologies by examining the mechanisms through which mHealth application usability influences users’ behavioral intentions in the context of Turkey’s national digital health platform, e‐Nabız. By integrating usability and e‐satisfaction perspectives, the study provides insights into how system design characteristics translate into behavioral outcomes in mHealth environments. In particular, the research extends prior studies on mHealth adoption by focusing on a nationally integrated digital health platform where system usage is largely unavoidable for citizens seeking access to healthcare services.

The findings related to H1a and H1b indicate that usability perceptions significantly influence users’ satisfaction with the mHealth application. The results show that positive use had a significant positive effect on e‐satisfaction, supporting H1a. This finding suggests that when users perceive the application as easy to use, integrated, and reliable, they tend to evaluate the system more positively. This result is consistent with previous studies highlighting the importance of usability in shaping user perceptions and satisfaction in mHealth services [64, 65]. Similarly, studies examining digital health and wearable technologies emphasize that usability and system quality are key determinants of positive user experiences and satisfaction [66].

However, the results did not support H1b, which predicted that negative use would negatively influence e‐satisfaction. Instead, a significant positive relationship was observed. This unexpected finding can be attributed to the quasimandatory nature of e‐Nabız: As a centralized national digital health infrastructure, users may tolerate usability challenges because the platform provides critical benefits such as access to medical records, prescriptions, and test results. Furthermore, the negative‐use construct was measured with reverse‐coded SUS items, which may reflect system sophistication and functional depth as well as difficulty. Consequently, higher negative‐use scores may indicate users’ awareness of the platform’s comprehensive functionality rather than dissatisfaction.

The results related to H2a and H2b demonstrate that e‐satisfaction plays a crucial role in shaping users’ behavioral intentions. The findings reveal that e‐satisfaction significantly and positively influences both recommendation intention and continued preference intention, supporting H2a and H2b. These results are consistent with satisfaction and continuance intention literature, which suggests that satisfied users are more likely to continue using a system and recommend it to others [22, 67]. Similar conclusions have been reported in recent studies examining mHealth and fitness applications, where satisfaction has been identified as a key determinant of continued usage intention [68, 69].

Building upon these findings, the mediation hypotheses (H3a–H3d) examined whether e‐satisfaction acts as a mechanism linking usability perceptions with behavioral intention. The results confirmed that e‐satisfaction mediates the relationship between usability dimensions and behavioral intention outcomes. The bootstrapping analysis showed that the indirect effects were significant and the confidence intervals did not include zero, supporting H3a, H3b, H3c, and H3d. These findings align with contemporary mediation analysis approaches, suggesting that mediation can be established when indirect effects are statistically significant [70, 71]. The results, therefore, highlight that usability influences behavioral intention primarily through users’ satisfaction with the application.

An important implication of this finding is that usability alone may not be sufficient to directly influence behavioral intention in the context of a national digital health system. Instead, usability first shapes users’ satisfaction with the system, which subsequently influences their intention to continue using and recommending the platform. This suggests that satisfaction operates as a key psychological mechanism translating system characteristics into behavioral outcomes.

Finally, the findings should also be interpreted in light of the quasimandatory nature of the e‐Nabız platform. Unlike many commercial mobile applications, where users can freely switch between alternative services, national digital health systems often function as centralized infrastructures with limited alternatives. Prior research on mandatory information systems suggests that users’ behavioral responses in such environments may differ from voluntary technology adoption contexts [72, 73]. Studies on e‐government technologies similarly highlight that citizens’ satisfaction plays a critical role in shaping their acceptance and continued use of mandatory digital services [74, 75]. In this respect, the present findings suggest that satisfaction becomes a critical mechanism linking system usability and behavioral intention in mandatory digital health environments.

### 5.1. Practical Implications

The findings of this study provide several practical implications for healthcare managers, policymakers, and digital health system developers. These implications are particularly relevant for nursing management, institutional governance, and national digital health leadership. As national digital health platforms increasingly become central components of healthcare delivery, understanding the factors that shape users’ behavioral intentions is critical for improving system adoption and long‐term engagement.

The results highlight user satisfaction as a key mechanism linking usability and behavioral intention. While usability perceptions did not consistently predict behavioral intention directly, they significantly influenced e‐satisfaction, which in turn affected users’ intentions to continue using and recommending the application. This finding aligns with information systems literature suggesting that satisfaction plays a crucial role in users’ continuance intentions [67]. Therefore, healthcare managers should focus not only on improving the technical usability of digital health platforms but also on enhancing overall user satisfaction. Nursing managers, in particular, can support patients in navigating mHealth applications effectively, guiding them to perceive the benefits of these systems and enhancing their engagement.

Policymakers responsible for national digital health platforms such as e‐Nabız should recognize that citizens’ continued engagement with these systems largely depends on their satisfaction with the quality, reliability, and usefulness of the services provided. National digital health authorities should prioritize governance strategies that strengthen public trust, transparency, and perceived value of digital health infrastructures, recognizing that satisfaction mediates engagement even when usability issues exist. Regular monitoring of user satisfaction and incorporating user feedback into system updates may significantly improve the effectiveness and sustainability of national digital health infrastructures.

Application developers should prioritize user‐centered design principles when designing and improving mHealth platforms. Ensuring that applications are intuitive, reliable, and responsive can strengthen positive user perceptions and increase satisfaction. Previous studies in the mHealth domain have also emphasized the importance of usability in shaping user experience and satisfaction with digital health services [64]. Healthcare organizations should also facilitate ongoing training and digital literacy programs for both staff and patients, ensuring that usability improvements translate into meaningful engagement at the clinical and institutional levels.

To enhance user satisfaction and encourage continued usage of national digital health platforms, healthcare organizations and developers may consider implementing the following strategies:

- Improve usability through user‐centered design: Application interfaces should be regularly evaluated and simplified to ensure that users can easily access health records, appointments, and other digital services.
- Strengthen user feedback mechanisms: Integrating feedback channels within the application can help developers quickly identify user concerns and continuously improve system performance.
- Increase the reliability and transparency of digital health services: Ensuring that health data are accurate, up‐to‐date, and securely stored may strengthen users’ trust and satisfaction with the platform.
- Support user guidance and digital literacy: Providing tutorials, help sections, and in‐app guidance can assist users who experience difficulties navigating digital health systems.

### 5.2. Limitations and Future Research Directions

This study has several limitations that should be acknowledged. The data were collected using a cross‐sectional survey design. Although statistical procedures were applied to reduce the potential risk of CMB, cross‐sectional data do not allow strong causal inferences to be drawn between the studied variables [47]. Future research may therefore employ longitudinal designs to better examine causal relationships between usability perceptions, satisfaction, and behavioral intentions over time.

Another limitation relates to the use of a convenience sampling approach for collecting data from e‐Nabız users. While this approach enabled the researchers to reach a relatively large sample, it may restrict the generalizability of the findings. Future studies could utilize probability‐based sampling techniques or nationally representative samples in order to enhance the external validity of the results. The contextual setting of the study also represents an important limitation. The research was conducted within the context of Turkey’s national digital health platform. Cultural differences may influence how users perceive and interact with digital technologies. Previous research suggests that cultural characteristics such as power distance and uncertainty avoidance may shape individuals’ attitudes toward technological systems and institutional platforms [76]. Replicating this study in different countries and healthcare systems could therefore provide valuable insights into whether similar relationships emerge across diverse cultural contexts.

Finally, the present study focused on usability perceptions and e‐satisfaction as determinants of behavioral intention. However, other important factors such as perceived usefulness, trust, privacy concerns, or digital health literacy may also influence users’ behavioral intentions toward mHealth applications. Future research may extend the current model by incorporating these additional variables to provide a more comprehensive understanding of user behavior in digital health platforms.

In conclusion, this study examined the relationships between mHealth application usability, e‐satisfaction, and behavioral intention within the context of Turkey’s national digital health platform, e‐Nabız. The findings highlight the important role of e‐satisfaction as a mediating mechanism linking usability perceptions to behavioral intention. By providing empirical evidence from a large‐scale national digital health system, the study contributes to the growing literature on user behavior in mHealth technologies and offers insights for improving the design and sustainability of digital health platforms.

## Funding

No funding was received for this manuscript.

## Disclosure

This article is derived from the doctoral thesis titled “The Mediating Role of e‐Satisfaction in the Effect of Mobile Health Application Usability on Behavioral Intention: The Case of e‐Nabız,” completed by Burhan Kılıç at Hasan Kalyoncu University, Turkey, in 2023.

## Conflicts of Interest

The authors declare no conflicts of interest.

## Data Availability

The data that support the findings of this study are available from the corresponding author upon reasonable request.

## Appendix 1 Confirmatory Factor Analysis.

**TABLE A1:** Confirmatory factor analysis.

Variables	Loading	*α*	CR	AVE	MSV
*Positive use*					
I think that I would like to use this application frequently.	0.522	0.764	0.770	0.580	0.473
I think this application is easy to use.	0.740				
I found the various functions in this application to be well integrated.	0.629				
I believe that most people would learn to use this application very quickly.	0.608				
I felt very confident while using this application.	0.659				

*Negative use*					
I found this application unnecessarily complex.	0.635	0.729	0.740	0.555	0.213
I think I would need the support of a technically skilled person to be able to use this application.	0.560				
I thought there was too much inconsistency in this application.	0.683				
I found this application very cumbersome to use.	0.684				
I needed to learn many things before I could effectively use this application.	0.441				

*e-satisfaction*					
I am satisfied with using this application to receive services.	0.779	0.876	0.880	0.801	0.582
If I need to perform similar transactions again, my perception of using this application will be positive.	0.799				
Choosing this application was a wise decision.	0.818				
I feel good about my decision to use this application.	0.806				

*Recommendation intention*					
I would say positive things about this application to people around me.	0.823	0.898	0.910	0.846	0.582
If asked for advice, I would recommend this application.	0.921				
I would encourage my friends and relatives to use this application for healthcare services.	0.857				
I feel proud to tell others that I receive services through this application.	0.777				

*Continued preference intention*					
If I need healthcare services again, I would consider this application as my first choice.	0.781	0.877	0.870	0.792	0.570
I intend to continue using this application for healthcare services in the coming years.	0.728				
I intend to use this application to meet all my healthcare‐related needs.	0.805				
If I had to make the choice again, I would choose this application.	0.848				
